# Personalized Approaches to Patients with Intra-Abdominal Infections

**DOI:** 10.3390/jcm14217774

**Published:** 2025-11-01

**Authors:** Massimo Sartelli, Federico Coccolini, Francesco M. Labricciosa, Walter Siquini, Giuseppe Pipitone, Miriam Palmieri, Valentina Sbacco, Carlo Vallicelli, Cristina Marmorale, Fausto Catena

**Affiliations:** 1 Department of General Surgery, Macerata Hospital, 62100 Macerata, Italy; walter.siquini@sanita.marche.it (W.S.); miriam.palmieri@sanita.marche.it (M.P.); valentina.sbacco@sanita.marche.it (V.S.); 2 General, Emergency and Trauma Surgery Department, Pisa University Hospital, 56124 Pisa, Italy; federico.coccolini@unipi.it; 3 Global Alliance for Infections in Surgery, 62100 Macerata, Italy; labricciosafrancesco@gmail.com; 4 Infectious Diseases Unit, ARNAS Civico-Di Cristina, 90127 Palermo, Italy; 5 General, Emergency and Trauma Surgery Department, Bufalini Hospital, 47521 Cesena, Italy; carlo.vallicelli@auslromagna.it (C.V.); fausto.catena@auslromagna.it (F.C.); 6 Department of Surgery, Università Politecnica delle Marche, 60126 Ancona, Italy; c.marmorale@univpm.it; 7 Department of Medical and Surgical Sciences, Alma Mater Studiorum University of Bologna, 40126 Bologna, Italy

**Keywords:** antimicrobial resistance, antimicrobial stewardship, antimicrobial therapy, intra-abdominal infections

## Abstract

Intra-abdominal infections (IAIs) continue to be an important cause of morbidity and mortality worldwide. The optimal management of patients with IAIs relies on early and accurate diagnosis, prompt and adequate source control, appropriate antimicrobial therapy based on the PK/PD principles, as well as hemodynamic support with intravenous fluids and vasopressors in critically ill patients. This narrative review aims to suggest five basic factors which should always be considered when assessing patients with IAIs to provide the most adequate treatment. These factors include the anatomical extent of the infection, the origin of the infection, the patient’s clinical status, the suspected pathogens and their resistance profiles, and immune competence. The continuous assessment of these elements is essential in managing complicated IAIs.

## 1. Introduction

Intra-abdominal infections (IAIs) continue to be an important cause of morbidity and mortality throughout the world. The mortality risk is frequently underestimated in clinical trials because of patient selection bias. Case series and trials often recruit patients with uncomplicated acute appendicitis or exclude severely ill patients and individuals with considerable comorbidities, explaining the fairly low mortality rates according to published studies [1]. Indeed, observational data from the real world suggest increased mortality rates [2], particularly in patients with multiple organ dysfunction syndrome. The WISS (WSES complicated IAIs Score Study) study, including 4553 patients with cIAI from 132 facilities all over the world, demonstrated a mortality rate of 9.2%, giving a more accurate picture of the clinical course [3]. The management of patients with cIAIs involves a meticulous assessment of the patient’s condition and the severity of illness, as well as appropriate interventions, including timely source control, adequate antimicrobial therapy, and hemodynamic support with fluids and vasopressors when required.

**Figure 1:**
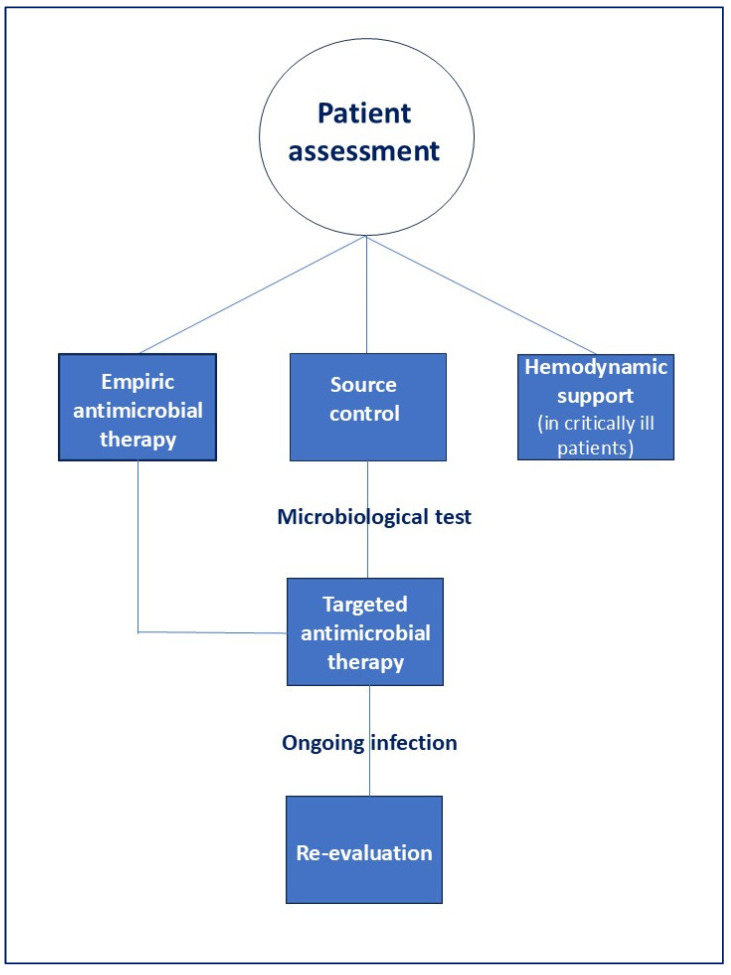
Correct management of patients with IAIs.

To optimize the treatment of IAIs, it is crucial to perform a comprehensive assessment of the patients. This narrative review aims to propose five fundamental factors that should always be evaluated during the assessment of patients with IAI to ensure that they receive the most appropriate treatment (Figure 1):

- the anatomical extent of the infection,
- the origin of the infection,
- the patient’s clinical condition and
- the presumed pathogens involved and risk factors for antimicrobial resistance,
- the host’s immune status.

**Figure 2:**
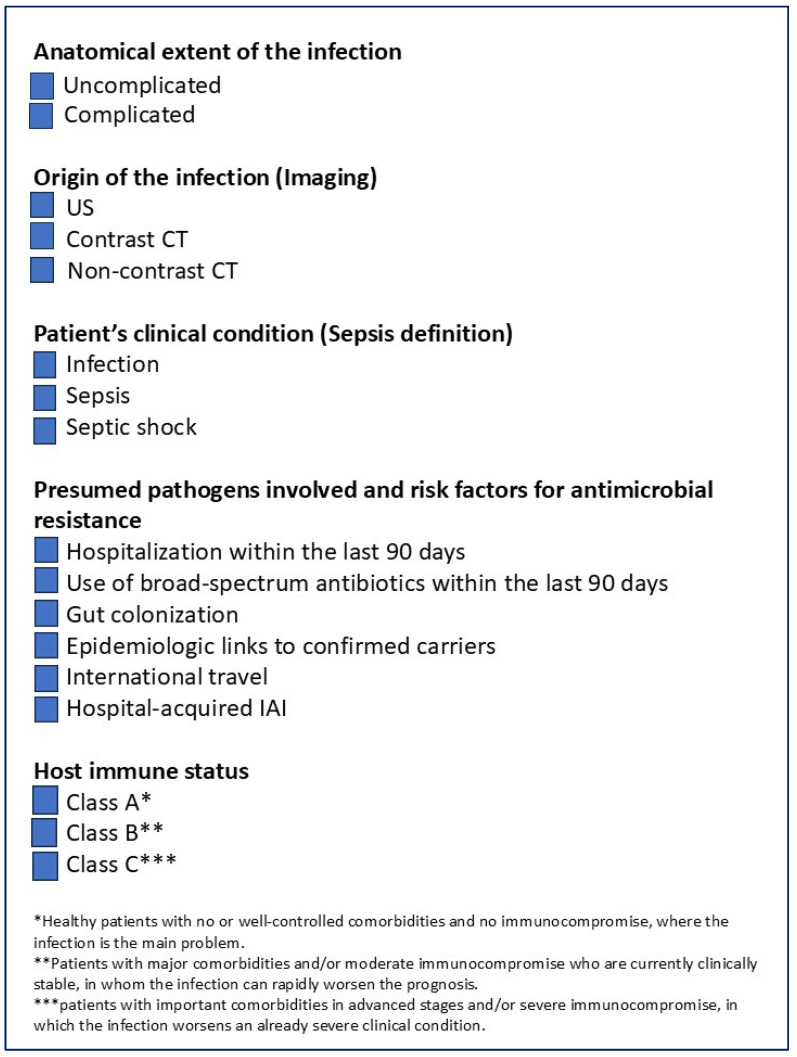
Checklist for the clinical assessment of patients with IAI.

A careful assessment of these factors is crucial to optimize the management of patients with IAIs (Figure 2).

## 2. Materials and Methods

This is a narrative review including articles from PubMed (Bethesda, MD, USA) and Google Scholar (Mountain View, CA, USA), published between 1 January 2010, and 31 December 2024, supplemented by earlier articles emphasizing timeless points. The following list of search terms was used: “antimicrobial resistance”, “antimicrobial therapy”, “intra-abdominal infections”. Articles in languages other than English were excluded. A total of 675 articles were retrieved.

Two authors screened titles and abstracts of gathered references for eligible articles. Full texts of all relevant papers were retrieved. Ultimately, a total of 120 references were considered as supporting evidence. Afterwards, the first draft was shared with the other authors, who independently reviewed the document and completed it. The resulting document was submitted again to all authors, reviewed, and finally approved.

## 3. Factors to Assess for Optimizing Outcomes in Patients with cIAIs

### 3.1. Origin of Infection

Both the diagnosis and the management of the source of infection are crucial for planning appropriate management in patients with cIAIs. The source control is defined by all procedures intended to remove the cause of infection and to prevent additional peritoneal contamination. The selection of the procedure should be individualized, based on the infection aetiology, patient characteristics, and local surgical experience. In a few cases, minimally invasive procedures such as ultrasonography (US)- or computed tomography (CT)-guided percutaneous drainage can be carried out. Efficient source control needs knowledge of the physiopathology of sepsis, host-immune response and surgical and non-surgical procedures [4]. The diagnosis of IAIs is essentially clinical. The patients usually come to the emergency department with abdominal pain and systemic inflammatory markers, such as fever, tachycardia, tachypnea and rising white blood cell count. The cIAI may be detected by abdominal tenderness. Laboratory investigations, including complete blood counts, are frequent but nonspecific. Biomarkers such as C-reactive protein and especially PCT can significantly contribute to the confirmation of bacterial infections [5,6].

US and CT are key diagnostic tools in IAIs. US is portable and can be performed at the bedside by surgeons or radiologists. US limitations include ileus and obesity; it is highly operator-dependent and is considered the radiological modality of first choice for children and adults with acute cholecystitis.

For most patients with cIAI, CT provides higher diagnostic accuracy compared to US, especially for identifying the source of infection in hemodynamically stable patients. In these patients, CT with intravenous contrast is the imaging modality of first choice [7,8,9,10]. Contrast-enhanced CT represents a standardized, operator-independent assessment evaluating multiple body regions quickly, identifying in most cases the source of infection. Contrast-enhanced CT can provide anatomical details of the intestinal wall, enabling the detection of the surrounding mesentery. Contrast-enhanced CT can also demonstrate segmental intestinal ischemia and extraluminal air within the peritoneal cavity. Furthermore, it can give essential information for planning an appropriate treatment strategy, helping clinicians select the most effective management pathway for each patient.

Source control plays a critical role in treating patients with cIAIs. It refers to all physical interventions aimed at eliminating the abdominal source of infection and restoring normal function. Non-surgical interventional options, such as percutaneous drainage guided by US or CT, can offer a less invasive, safe, and effective approach for treating intra-abdominal and extra-peritoneal abscesses in selected patients.

Surgical source control for IAIs may involve resecting or suturing a diseased or perforated portion of the gastrointestinal tract (e.g., diverticular peritonitis or gastroduodenal perforation), removing an infected organ (such as the acute appendicitis or acute cholecystitis), debriding dead tissue, excising ischemic bowel, or repairing/resecting traumatic perforations, which may be followed by primary anastomosis or creation of a stoma.

Rapid and precise source identification is critical in managing critically ill patients, and delays in surgical source control (i.e., >6 h from sepsis onset) are associated with worse outcomes [11]. In severely physiologically unstable patients, early surgical exploration may be warranted even if imaging fails to identify the source of infection [12].

### 3.2. Anatomical Extent of the Infection

Understanding the extent of the infection is a critical point in assessing patients with IAIs. A universally accepted classification of IAIs divides them into uncomplicated IAIs (uIAIs) and cIAIs.

In patients with uIAIs, the infectious process only involves a single organ and does not proceed to the peritoneum. In patients with cIAIs, the infectious process extends into the peritoneum, causing localized or diffuse peritonitis. Although this classification does not identify patients based on their complexity, it helps plan the treatment strategy. Patients with uncomplicated appendicitis and cholecystitis can be managed by surgical intervention or antibiotics alone [13,14,15,16]. In these patients, post-operative antibiotics are unnecessary if adequate source control is achieved [17,18,19]. Although the “first-choice” treatment of acute appendicitis is laparoscopic appendectomy [20], non-operative management of uncomplicated appendicitis is now considered safe and effective. However, it may be associated with higher recurrence (up to 40%) [17,18,19] and the potential risk of perforation, particularly when preoperative delays occur without an accurate CT-based diagnosis of uncomplicated appendicitis.

In the management of acute cholecystitis, surgical intervention remains the first-line treatment. For acute, uncomplicated cholecystitis, two strategies are available. Early laparoscopic cholecystectomy is performed within a few days of symptom onset during the initial hospital admission following confirmation of diagnosis and offers an immediate, definitive treatment. Alternatively, delayed laparoscopic cholecystectomy is performed during a subsequent admission, typically 6–12 weeks later, once the acute inflammatory process has resolved.

In patients with acute uncomplicated cholecystitis, early laparoscopic cholecystectomy within a week of symptom onset is generally preferred because it reduces hospital stay compared to delayed surgery [21]. A meta-analysis conducted by Lyu et al. [21], encompassing 15 randomized controlled trials and enrolling 1669 patients, demonstrated that early laparoscopic cholecystectomy is as safe and effective as delayed cholecystectomy when it is performed within 7 days of presentation for patients with acute cholecystitis. No significant differences were observed between the two approaches regarding bile duct injuries, wound infections, overall complications, or conversion to open surgery. Moreover, pooled analyses indicated that early surgery was associated with a significantly shorter total hospital stay, even if the postoperative length of stay did not differ significantly.

Several randomized trials demonstrated that uncomplicated acute diverticulitis in stable patients can be managed without antibiotics [22,23,24,25]. The DIABOLO trial found similar complication rates between observational and antibiotic-treated groups, demonstrating shorter hospital stays in the observation group [24]. The outcomes of patients in the DIABOLO study were also analyzed at a 24-month follow-up [26]. Analyses of the cases revealed no significant differences in the incidence of recurrent diverticulitis, complicated diverticulitis, or the need for sigmoid resection.

On the contrary, treatment of patients with cIAIs requires both source control and antibiotic therapy. Evidence supports a short duration of antibiotic therapy when source control is adequate [27]. The STOP-IT trial demonstrated that patients treated with four-day antibiotic therapy after effective source control had similar outcomes compared to longer courses, even in high-risk patients [28]. Retrospective analysis of the STOP-IT trial data evaluated risk factors associated with treatment failure [29], including corticosteroid use, an APACHE-II score of ≥5, hospital-acquired infections, and a colonic source of cIAI. Despite these risk factors, treatment failure rates did not differ between the groups, suggesting that extended-duration therapy offers no further benefits even in high-risk patients.

Postoperative antibiotic duration can potentially be shortened in complicated appendicitis, as demonstrated by recent studies showing no difference in outcomes between 2-day and 5-day regimens [30].

Critically ill patients often receive longer antibiotic courses. The DURAPOP trial [31] demonstrates that therapy can be safely reduced in critically ill patients with post-operative peritonitis. Patients who received 8 days of treatment had more antibiotic-free days compared to those treated for 15 days. Mortality at 45 days was equivalent between groups (rate difference 0.038, 95% CI −0.013 to 0.061). No differences were found in intensive care unit (ICU) or hospital stay duration, development of multidrug-resistant bacteria, or reoperation rates.

Some patients remain at risk of persistent signs of an ongoing infection. These patients should always warrant diagnostic reassessment to identify unresolved sources of infection and the need for a re-laparotomy. In such cases, timely re-laparotomy can be a critical surgical option.

Procalcitonin (PCT) is a useful biomarker to guide antibiotic therapy [32,33,34,35,36], allowing for shorter treatment courses of antibiotics. Many studies indicate that PCT-guided therapy in cIAI patients reduces antibiotic duration without increasing complications or mortality [37,38,39].

### 3.3. Clinical Conditions

The early identification of sepsis and the prompt treatment can significantly improve patient outcomes. However, the diagnosis of sepsis in its early stages is challenging, as it’s often difficult to determine whether a patient showing signs of infection will progress to a more critical condition. Sepsis is a multifaceted condition with varying degrees of severity. If not treated promptly, it may cause dysfunction of one or more vital organs, with the risk of mortality rising as the condition advances.

Information from the WISS study demonstrates that sepsis significantly increases mortality, especially in the presence of organ dysfunction. In the study, reported mortality rates were: no sepsis 1.2%, sepsis 4.4%, severe sepsis 27.8% and septic shock 67.8% [3]. Sepsis definitions have now evolved with SEPSIS-3. Sepsis is now defined as an infection with organ dysfunction and eliminating “severe sepsis” [40,41]. Organ failure is defined as an increase in the Sequential Organ Failure Assessment (SOFA) score of ≥2 points, and septic shock is indicated by a vasopressor requirement to maintain mean arterial pressure (MAP) > 65 mmHg and lactate > 2 mmol/L after fluid resuscitation [42]. Prompt identification and management, including resuscitation, antibiotic therapy, and source control, can improve patients’ outcomes [43,44].

Prompt intravenous fluid therapy is essential to manage patients with sepsis and septic shock. Studies comparing balanced crystalloids with saline suggest that balanced crystalloids can reduce mortality and renal complications [45,46]. Although albumin may better maintain oncotic pressure, it is costlier and does not offer any routine benefit. It is generally considered when large fluid volumes are required [44]. If fluid resuscitation is insufficient, vasopressors should be used to maintain organ perfusion [47]. Norepinephrine is the first-line vasopressor for patients with septic shock, exerting primarily beta-adrenergic effects at low doses and alpha-adrenergic effects at higher doses [47,48]. While most patients respond, a notable proportion demonstrate poor catecholamine responsiveness and require high doses (>0.5 mcg/kg/min) to achieve a MAP of 65 mmHg, when patients fail to reach this target despite maximal vasopressor support and optimized fluid therapy [49], second-line vasopressors may be preferable to further escalating norepinephrine. Low-dose vasopressin (0.03–0.06 UI/min continuous infusion) [50] can reduce mortality in less severe septic shock and provides a catecholamine-sparing effect, lowering norepinephrine requirements when used in combination.

Therefore, if patients require a dose of norepinephrine >0.25 mcg/Kg/min, adding vasopressin should be suggested, even if, when administering vasopressin, it is mandatory to have caution because of the potential ischemia of limb extremities.

In adults with sepsis or septic shock of abdominal origin, avoiding fluid overload is crucial to prevent poorer outcomes. Excessive fluid resuscitation, especially in patients requiring emergent surgical intervention, can increase intra-abdominal pressure (IAP), exacerbating the inflammatory response, elevating the risk of complications [51]. From a pathophysiological perspective, systemic inflammation, heightened vascular permeability, and large volumes of crystalloids can contribute to fluid sequestration. In advanced sepsis, bowel oedema from bowel shock and forced abdominal closure further increase IAP, potentially leading to intra-abdominal hypertension (IAH).

Early empiric antibiotics are essential, especially among patients in septic shock, because in these individuals, delayed therapy increases death [52]. Given the high risk of mortality in patients with septic shock and the strong association between timely antibiotic administration and survival, it is crucial to administer antibiotics immediately in all patients with septic shock. The impact of time to antibiotics on mortality in the first few hours of presentation is less pronounced among sepsis patients without shock [53,54]. Therefore, early aggressive use of antibiotics is recommended in these patients once sepsis is highly suspected.

When treating severely ill patients with sepsis or septic shock, clinicians should be aware that antibiotic PK may be altered. According to the ‘dilution effect’, patients need beta-lactams in doses higher than conventional ones, even when there is no marked impairment of renal function, if we aim at concentrations in the site of infection [55]. Once therapy is initiated, daily reassessment is critical as shifts in fluid balance and organ function may impact drug PK. It should be noted that plasma creatinine is not a reliable marker of renal function in this patient subset. Appropriate dosing is based on the antibiotic’s bactericidal effect, which is time-dependent or concentration-dependent. Beta-lactam agents, which are time-dependent, act optimally when their concentrations remain just above the pathogen’s MIC for at least 70% of the dosing interval [56]. Prolonged or continuous infusions of beta-lactam agents can maintain concentrations above the MIC for longer periods [57,58,59] and are generally suggested, especially in critically ill patients.

Conversely, antibiotics with concentration-dependent activity achieve the maximal activity when the peak plasma concentration (Cmax) to MIC ratio (Cmax/MIC) exceeds 8–10 [55]. Therefore, once-daily pulse dosing is generally the preferred administration method for these antibiotics. For aminoglycosides, once-daily dosing reduces the risk of nephrotoxicity compared with multiple daily dosing. This benefit arises because accumulation in the renal cortex, mediated by specific carriers, can be more effectively saturated with a single high-dose administration, thereby limiting overall renal exposure.

Tissue penetration is also crucial, because adequate antibiotic levels at the site of infection contribute to minimize resistance. In general, lipophilic antibiotics penetrate tissues better than the hydrophilic ones; however, distribution is condition-dependent and may be modified by variables such as plasma protein binding and the state of disease. Consequently, patients with severe IAIs may require higher doses of beta-lactam to achieve therapeutic tissue concentrations [60].

TDM is important to optimize and personalize antibiotics dosing, yet TDM cannot be performed in all centers for practical reasons based on the necessity of specialized infrastructure, skills/knowledge [61]. The decision to accurately obtain source control in the sepsis patient often requires the knowledge of sepsis pathophysiology, available surgical and nonsurgical options and the ability to weigh risks versus benefits.

Source control should be performed as promptly as possible after initial resuscitation, with limited evidence suggesting that interventions within six hours yield the best outcomes. The results of a study by Bloos et al., published in 2014, enrolling 1011 patients in 44 German ICUs, demonstrated that the median time to source control was 2 h for survivors and 5.7 h for non-survivors. The study demonstrated that delaying six hours in controlling the source of infection was independently linked to higher mortality, along with factors such as age and illness severity [62]. In 2017, the results of a randomized controlled study found that the delay in surgical source control was strongly associated with 28-day mortality, and each hour of delay increased mortality by 1% [63]. Similarly, a prospective study, published in 2014, enrolling 154 patients with gastrointestinal perforation, showed that survival decreased with each hour of delay, emphasizing a six-hour target for optimal outcomes [64].

A 2022 post hoc analysis of the multi-center Abdominal Sepsis Study (AbSeS), which included 2621 ICU patients with cIAIs involving 306 ICUs in 42 countries, reported a mortality rate of 29.7% in 1077 patients with microbiologically confirmed secondary peritonitis [65]. Mortality increased progressively with higher SOFA scores, and the highest risk of death was associated with septic shock, late-onset hospital-acquired peritonitis, and failed source control. Interestingly, compared to emergency source control (<2 h from the presentation), urgent interventions were the only modifiable factor associated with lower mortality.

The most robust evidence supporting the six-hour target comes from a post hoc analysis of the MEDUSA trial [66]. This study enrolled 4792 patients receiving antimicrobial therapy and 1595 undergoing surgical source control in 40 German hospitals. Mortality rose by 0.42% per hour of delay, significant for both patients with and without shock. Delays in source control beyond six hours markedly increased mortality, and each hour of delayed antibiotic therapy raised the risk of progression from sepsis to septic shock. After adjusting for confounders, time to surgical source control did not affect the likelihood of successful intervention or overall mortality, except in patients with septic shock, where delays were linked to worse outcomes.

Following its success in trauma care, damage control laparotomy has been applied to non-traumatic emergencies, including critically ill patients with cIAIs [67]. In the context of sepsis of abdominal origin, damage control laparotomy, defined as a brief initial laparotomy followed by delayed definitive repair after physiologic stabilization [68], can be life-saving. This approach is closely associated with the “open abdomen” technique, which allows for manual peritoneal cleansing while intraperitoneal defences recover from the infection.

The open abdomen, maintained with a temporary closure device that can be easily removed or replaced, enables early detection and drainage of residual infection and removal of peritoneal fluid, reducing the risk of abdominal compartment syndrome. Definitive repair, including anastomosis, is postponed until the patient is stabilized, and peritoneal contamination is controlled. However, open-abdomen strategies often require multiple returns to the operating room or bedside interventions in the ICU and can carry significant risks, such as entero-atmospheric fistulas, loss of abdominal wall integrity, and large hernias. This highlights the importance of early abdominal wall reconstruction, ideally within seven days, to mitigate these complications. The ongoing COOL study may provide further guidance on the role of open abdomen management in abdominal sepsis [69].

### 3.4. Presumed Pathogens Involved and Risk Factors for Antimicrobial Resistance (For the Correct Antimicrobial Selection)

Understanding the patient risk factors for difficult-to-treat organisms is essential for starting an appropriate empiric antibiotic therapy, especially in an era of antimicrobial resistance (AMR) [70]. Initial treatment of cIAIs is generally empiric because microbiological testing and susceptibility results usually require 24–72 h after collection of peritoneal fluid. The bacteria commonly responsible for community-acquired IAIs typically originate from the patient’s gut flora, including *Enterobacterales* such as *Escherichia coli* and *Klebsiella* species, viridans group *Streptococcus*, and anaerobes, particularly *Bacteroides* species [4]. Bacteria isolated in patients with community-acquired IAIs generally show higher antibiotic susceptibility compared with those isolated in patients with hospital-acquired infections.

Patients with community-acquired IAIs can be treated with beta-lactam/beta-lactamase inhibitor combinations, such as amoxicillin/clavulanate, ticarcillin/clavulanate, piperacillin/tazobactam, or non-pseudomonal carbapenems such as ertapenem. However, the high levels of resistance to amoxicillin/clavulanate among *E. coli* and other *Enterobacterales* can limit its empiric use, and local resistance epidemiology should guide its prescription [4]. Among beta-lactam/beta-lactamase inhibitor combinations, piperacillin/tazobactam retains broader-spectrum activity and remains a viable option for patients with severe cIAI. However, its anti-pseudomonal effects are usually unnecessary in community-acquired cases. In hospital-acquired cases, its use should be evaluated according to local hospital epidemiology [71].

Third-generation cephalosporins, which should always be combined with metronidazole to cover anaerobes, in treating patients with IAIs, remain effective against many *E. coli* and *Enterobacterales* strains in patients with non-severe IAIs. Fourth-generation cefepime is less inactivated by AmpC beta-lactamases and, like third-generation cephalosporins, should be used in combination with metronidazole for treating patients with IAIs. Fluoroquinolones have historically been used due to their strong activity against Gram-negative bacteria and good tissue penetration; however, global resistance in *E. coli* limits their empirical use also in patents with community-acquired infections [72].

In 2019, a multinational cohort study of ICU patients with IAIs was published. It describes the epidemiology of IAIs according to the setting of infection acquisition (community-acquired, early-onset hospital-acquired, and late-onset hospital-acquired), anatomical disruption (absent or present with localized or diffuse peritonitis), and severity of disease expression (infection, sepsis, and septic shock).

The study reported 31.6% of patients with community-acquired and 68.4% of patients with hospital-acquired infections (early-onset hospital-acquired in 25%, and late-onset hospital-acquired in 43.4% of patients) [73]. Overall mortality was 29.1%. Gram-negative bacteria were isolated in 58.6% of cases, mainly *Enterobacterales* (51.7%). Gram-positive aerobes were found in 39.4% of cases, and MDR bacteria were common (26.3%), without major differences between community- and hospital-acquired infections.

In recent years, AMR has emerged as a global burden. The rise in infections caused by resistant Gram-negative bacteria poses an escalating threat to public health worldwide. These infections are challenging to treat and are associated with elevated morbidity and mortality rates.

In an era of AMR, it is very important to identify patients at high risk of colonization or infection by resistant bacteria, especially in critically ill patients who require early targeted therapy. Standardized definitions for acquired AMR have been well defined and include [74]:

- Multi-drug resistance (MDR): non-susceptibility to at least one agent in three or more antibiotic classes.
- Extensively drug-resistant (XDR): non-susceptibility to all but one or two antibiotic classes.
- Pan-drug resistance (PDR): non-susceptibility to all antibiotics in all classes.

Previously, the prediction of MDR bacteria was based on the setting of infection acquisition, whether infections were community- or hospital-acquired. However, the rising rates of extended-spectrum beta-lactamases (ESBLs)-producing *Enterobacterales* and carbapenemase-producing *Enterobacterales* (CPE) isolated in patients with community-acquired infections have complicated the empiric therapy selection also in the setting of IAIs [72]. Risk factors for MDR bacteria include prior colonization, recent antibiotic exposure, comorbidities, impaired functional status, recent invasive procedures [75], and international travel [76]. The screening of patients for carbapenem-resistant *Enterobacterales* (CRE) is now recognized as a crucial measure for correct infection prevention and control [77]. Screening for CRE is generally recommended in patients with prior CRE colonization or infection, recent hospitalizations, repeated hospital treatments, epidemiologic links to confirmed carriers, or admission to high-risk units.

In the context of IAIs, ESBLs represent the main resistance concern.

ESBLs are enzymes that can hydrolyse and inactivate a wide variety of beta-lactam agents, including first-, second-, and third-generation cephalosporins, penicillins, and aztreonam.

The main risk factors for ESBL-producing infections are:

(1) hospitalization within the last 90 days,
(2) use of broad-spectrum antibiotics for 5 days within the last 90 days,
(3) gut colonization by ESBL within 90 days,
(4) patients coming from healthcare settings with a high incidence of MDR bacteria (e.g., elderly people living in long-term facilities)

They are common in hospital-acquired infections, but also frequently reported in community-acquired cases [71,78].

For patients at risk of infection with ESBL-producing *Enterobacterales,* especially those who are hemodynamically unstable, empiric antibiotic therapy with ESBL coverage should always be recommended. However, in non-critically ill patients, evidence does not consistently show a significant survival advantage from empiric anti-ESBL therapy [79].

Carbapenems remain the preferred treatment of ESBLs. Group 1 carbapenems (ertapenem) and Group 2 (imipenem/cilastatin, meropenem, doripenem) have similar coverage profiles against ESBLs [80]. Unlike ertapenem, Group 2 antibiotics also cover *Pseudomonas aeruginosa*. Additionally, imipenem/cilastatin, differently from meropenem and doripenem, has activity against enterococci that remain susceptible to ampicillin.

The use of piperacillin/tazobactam for treating ESBL-producing *Enterobacterales* has been debated and remains controversial [72]. Firstly, Gram-negative bacteria can simultaneously express ESBLs and AmpC beta-lactamases, along with other antibiotic resistance mechanisms, reducing the activity of piperacillin/tazobactam [76]. Secondly, its activity is also affected by the “inoculum effect,” as the minimum inhibitory concentration (MIC) rises significantly if a large bacterial load is present [76]. The MERINO trial, published in 2018 and conducted in 379 patients with bloodstream infections caused by ESBL-producing *Enterobacterales,* demonstrated that patients treated with piperacillin-tazobactam did not experience a non-inferior 30-day mortality, compared with patients treated with meropenem. These findings do not support the use of piperacillin-tazobactam in this setting [81]. A secondary study to evaluate the relationship between piperacillin/tazobactam and meropenem MICs, the presence of beta-lactam resistance genes, and mortality in the MERINO trial was published in 2021. After removing strains that were not susceptible, the 30-day mortality difference observed in the MERINO trial for piperacillin/tazobactam was less significant, demonstrating that isolates carrying both ESBL and OXA-1 genes exhibited higher piperacillin/tazobactam MICs and were linked to the greatest increase in 30-day mortality [82]. Even if piperacillin/tazobactam is not considered the antibiotic of first choice for treating ESBL-producing *Enterobacterales*, it may represent an interesting option in patients with cIAIs when adequate source control is performed and when the bacteria are fully susceptible (MIC ≤ 4 mg/L) [72]. In critically ill patients, especially those with hemodynamic impairment, high doses of piperacillin/tazobactam are always recommended to optimize the pharmacokinetic/pharmacodynamic (PK/PD) parameters [83].

Tigecycline remains a useful option for patients with complicated IAIs due to its favourable in vitro activity against anaerobes, enterococci and ESBLs, and to the high concentration achieved in the biliary tract [84]. However, in numerous trials, excess mortality was seen in patients managed with tigecycline when compared with other agents; in 12 of 13 phase 3 and 4 comparative clinical trials [85], all-cause mortality was found to be higher in the tigecycline group versus the comparison group. Study-level and patient-level analyses identified that patients in the hospital-acquired pneumonia trial, particularly those with ventilator-associated pneumonia with baseline bacteraemia, were at a higher risk of clinical failure and mortality. A mortality analysis was used to investigate the association of baseline factors in intra-abdominal infections, including severity of illness at study entry and treatment assignment, with clinical failure and mortality. Mortality modelling identified multiple factors associated with death, which did not include tigecycline [85]. Because of its high concentration in the biliary tract, despite its low performance in bacteraemia patients, tigecycline could be considered to treat patients with IAIs in combination with other antibiotics, when a secondary bloodstream infection is suspected.

Aminoglycosides are effective against aerobic Gram-negative bacteria and act synergistically against certain Gram-positive bacteria. They are active against *Pseudomonas aeruginosa*; however, they are ineffective against anaerobic bacteria. Due to their serious toxic side effects, the poor penetration in the ascitic fluid and the loss of bactericidal activity in the presence of acidic pH, aminoglycosides are not used for the routine empiric treatment of IAIs.

Fosfomycin is a broad-spectrum agent effective against multidrug-resistant bacteria, acting irreversibly by blocking the synthesis of the bacterial cell wall. Importantly, fosfomycin can work synergistically when combined with other antibiotics, targeting different bacterial pathways [86] and reducing antibiotic-associated toxicity. Due to its strong ability to penetrate tissues, fosfomycin, in association with other antibiotics, may be useful for treating patients with cIAIs complicated by MDR bacteria [86].

Eravacycline is a fluorocycline agent structurally related to tigecycline and offers a broader-spectrum activity, excluding, like tigecycline, *P. aeruginosa* [87,88]. In two randomized clinical trials (IGNITE 1 and 4), eravacycline demonstrated non-inferior clinical cure rates compared to ertapenem and meropenem at the test-of-cure visits (IGNITE 1: 87.0% vs. 88.8%; IGNITE 4: 90.8% vs. 91.2%) [48,49]. Exhibiting antibacterial effects against carbapenem-resistant Gram-negative bacteria, eravacycline has a potential role in the clinical management of patients with MDR bacterial IAIs. Additionally, eravacycline treatment carries a very low risk of *Clostridioides difficile* infection [89].

Both ceftolozane/tazobactam and ceftazidime/avibactam (CAZ-AVI) are effective options for treating cIAIs caused by ESBL-producing *Enterobacterales* [90,91], particularly in critically ill patients or when isolates exhibit high MICs, and have been proposed as options for a carbapenem preserve strategy.

A major global problem is the spread of carbapenem-resistant *Enterobacterales* (CRE) [92]. Carbapenemases are beta-lactamase enzymes that hydrolyse penicillins, all cephalosporins, first-generation beta-lactamase inhibitors, and carbapenems.

Several prospective studies support CAZ-AVI in the treatment of patients with cIAIs caused by *Klebsiella pneumoniae* carbapenemases (KPC). Other antibiotics targeting KPC include meropenem/vaborbactam and imipenem-cilastatin/relebactam, even if these antibiotics have not yet been specifically studied in cIAIs [93]. In the phase 3 RESTORE-IMI 1 trial, imipenem-relebactam, active against *Klebsiella pneumoniae* carbapenemase (KPC)-producing bacteria, was compared to imipenem plus colistin for hospital-acquired or ventilator-associated pneumonia, cIAIs, and complicated urinary tract infections caused by imipenem-non-susceptible organisms. Overall response rates were similar, but imipenem-relebactam showed better outcomes in patients with *P. aeruginosa*. Only two patients per arm with cIAIs were included. At day 28, favourable clinical responses and reduced all-cause mortality were higher in the imipenem-relebactam group, while serious adverse events, including nephrotoxicity, were more frequent with imipenem plus colistin [94]. Both meropenem-vaborbactam and imipenem-relebactam are effective against most KPC-producing *Enterobacterales* but lack activity against OXA-48-like carbapenemases.

Metallo-beta-lactamases (MBLs) are distinct from other beta-lactamases because they are zinc (Zn^2+^)-dependent enzymes. They can hydrolyze nearly all beta-lactam antibiotics, including carbapenems, except aztreonam. Treatment options for MBL-producing *Enterobacterales* include ceftazidime/avibactam combined with aztreonam or cefiderocol, even if cefiderocol has not yet been specifically studied for cIAIs. Cefiderocol is a cephalosporin that bypasses three key carbapenem-resistance mechanisms. It avoids porin channel and efflux pump limitations by entering bacterial cells through iron transport systems, and it remains stable against all four classes of beta-lactamases [95].

The aztreonam/avibactam combination is a novel antibiotic showing potential activity against difficult-to-treat MDR Gram-negative bacteria, including metallo-beta-lactamase-producing *Enterobacterales*, although it has not been specifically evaluated in patients with cIAIs [93].

In recent years, high rates of resistance have also been reported in non-fermenting Gram-negative bacteria, including *Pseudomonas aeruginosa*, *Stenotrophomonas maltophilia*, and *Acinetobacter baumannii*. They are intrinsically resistant to many antibiotics and can acquire additional resistance to other important antibiotic agents [76].

Therapeutic alternatives for infections caused by A. baumannii are scarce, particularly for those involving carbapenem-resistant *A. baumannii*. Two agents, such as cefiderocol and sulbactam-durlobactam, hold promise for the treatment of carbapenem-resistant *A. baumannii*, providing new useful options in this challenging clinical scenario.

Among Gram-positive bacteria, Enterococci are commonly isolated in IAIs [96,97]; however, the influence of these organisms on morbidity and mortality is unknown. Antibiotic therapy against Enterococci is not generally required for patients with community-acquired IAIs, but it is usually indicated for patients with hospital-acquired IAIs, immunosuppression or prosthetic devices [98,99]. In 2021, Zhang et al. published a meta-analysis which analyzed the efficacy of appropriate empiric therapy against Enterococci in patients with IAIs. The meta-analysis found that anti-enterococcal treatment did not impact treatment success in patients with cIAI, and no differences were found for mortality or adverse events, especially in randomized controlled trials involving younger individuals with low-risk community-acquired IAIs (median APACHE-II score ≈ 6) [99]. Malignancy, corticosteroid administration, previous operation, any preoperative antibiotic exposure, ICU admission and indwelling urinary catheters were all found as independent risk factors associated with the development of enterococcal infection in cIAI patients. Enterococcal isolation was reported to be 2–5 times higher in patients with hospital-acquired IAIs. *E. faecalis* is usually susceptible to ampicillin; however, *E. faecium* can show ampicillin resistance, and about 70% are resistant to vancomycin [72]. First-line therapy for vancomycin-susceptible *E. faecium* is vancomycin. Vancomycin-resistant *E. faecium* (VRE) can be treated with linezolid or daptomycin, although rare resistance to these agents has been reported. Glycylcyclines such as tigecycline and eravacycline also provide effective activity against VRE.

Although bacteriological testing has not traditionally been suggested for the treatment of patients with community-acquired cIAIs, such as appendicitis [100,101], the rise in MDR bacteria in both community-and hospital-acquired infections makes microbiological testing increasingly important. For patients at risk of MDR bacteria, peritoneal fluid microbiological cultures should always be collected, because early initiation of targeted antibiotic therapy is crucial for improving outcomes, making rapid diagnostic testing a key priority in tackling infections, particularly in critically ill patients [102].

Very importantly, microbiological cultures also provide valuable epidemiological data, helping to understand the local microbiological epidemiology.

The role of *Candida* species in patients with IAIs remains controversial and debated. Intra-abdominal candidiasis is rare, but it can cause high mortality, especially in critically ill patients. Risk factors include gastrointestinal perforation, anastomotic leakage, and prior antibiotic or antifungal exposure [103]. Empiric antifungal therapy is usually reserved for high-risk patients, including those with septic shock or immunocompromised status.

Echinocandins are recommended as first-line therapy for invasive candidiasis [104,105,106]. However, their use has been debated due to emerging antifungal resistance and suboptimal exposure in critically ill patients with IAIs, which may necessitate dose adjustments guided by therapeutic drug monitoring (TDM), because of their low penetration into the peritoneum [107,108]. Welte et al. assessed the pharmacokinetic profiles of anidulafungin, micafungin and caspofungin in plasma and ascitic fluid of 29 critically ill patients with intra-abdominal candidiasis. Standard dosages restricted the proliferation of *C. albicans* and *C. glabrata* in ascites but failed to eliminate them [109].

Literature data concerning isavuconazole penetration into ascitic fluid in critically ill patients were reported with contradictory observations, and treatment success appears to be dependent on *Candida* species susceptibility, host immune status and other parameters which warrant further study [110].

Azoles are no longer the preferred choice of antifungal agents for critically ill patients because of high resistance rates and the risk of drug interactions. An effective alternative is represented by liposomal Amphotericin B, a lipid-based form of amphotericin B [98]. The Liposomal Amphotericin B permits high dosing, offering better antifungal efficacy with fewer side effects and nephrotoxicity. This is linked to low potential for drug interactions and resistance development. It was effective against fungi in a dose-dependent manner, with a long half-life and in vitro sustained antifungal activity [111]. Its lipophilic character may also be less affected by physiological states than that of echinocandins. There is a lack of clinical data on its use for patients with IAIs [98]. However, its usage appears logical, and it has been considered to be an attractive option because of its acceptable safety profile in intensive care patients [98]. A single-center experience also supported the safety and cost-effectiveness of giving a 5 mg/kg loading L-AmB dose while waiting for 1,3-β-d-Glucan results [112].

### 3.5. Host Immune Status

The immune status of a patient plays a critical role in infection outcomes but is often difficult to quantify. Immunocompromised individuals represent a diverse population. Immunodeficiency can be congenital, such as T- or B-cell defects or macrophage dysfunction, affecting patients from infancy to adulthood. Acquired immunodeficiencies include HIV/AIDS, malignancies treated with chemotherapy, solid-organ transplant recipients, or patients receiving immunomodulatory therapy for inflammatory or rheumatologic conditions [113,114]. Rarely, immune checkpoint inhibitor therapy has been linked to acute appendicitis [115].

Diagnosing and treating IAIs in immunocompromised patients is challenging. These patients face higher morbidity and mortality [116,117,118] and often do not respond to standard non-operative management, making surgical intervention frequently necessary. Recent multi-society guidelines [119] recommend categorizing patients based on their clinical condition, comorbidities, ongoing therapies (e.g., anticoagulants or steroids), and immune status to guide source control.

Solid-organ transplantation has become a viable option for patients with end-stage organ failure. Advances in surgical techniques, perioperative care, immunosuppressive regimens, and infection prophylaxis have improved survival. While post-transplant cytomegalovirus infections have decreased, MDR Gram-negative infections are rising, reflecting frequent antibiotic exposure in healthcare settings [120]. This susceptibility to MDR pathogens is a key factor how initiating empiric antimicrobial therapy in solid-organ transplant recipients.

In 2023, a set of multi-society guidelines for source control in emergency surgery was published, proposing a system of patients’ stratification to be performed according to their current conditions, their comorbidities and ongoing therapies together with their immunological state.

Patients were categorized into three classes [114]:

- Class A includes healthy patients with no or well-controlled comorbidities and no immunocompromise, where the infection is the main problem.
- Class B includes patients with major comorbidities and/or moderate immunocompromise who are currently clinically stable, in whom the infection can rapidly worsen the prognosis.
- Class C includes patients with important comorbidities in advanced stages and/or severe immunocompromise, in which the infection worsens an already severe clinical condition.

## 4. Conclusions

IAIs are a leading cause of illness and death associated with hospital care across the globe. Effective management of cIAIs relies on prompt and precise diagnosis, timely and adequate control of the infection source, appropriate antimicrobial therapy tailored according to pharmacokinetic/pharmacodynamic principles and antimicrobial stewardship, as well as hemodynamic and organ support through intravenous fluids and vasopressors for critically ill patients (e.g., sepsis or septic shock with refractory hypovolemia).

Treatment for patients with cIAIs should be highly individualized, taking into account the infection’s anatomical extent, the infection origin, the patient’s clinical status, the suspected pathogens and their resistance profiles, and immune competence. Careful assessment of these factors is essential to optimize outcomes for patients with cIAI. In Figure 2, a simple checklist that every clinician should evaluate during the clinical assessment of patients with IAI is illustrated.

## Data Availability

Not applicable.

## Abbreviations

The following abbreviations are used in this manuscript:

AMR	Antimicrobial resistance
cIAI	Complicated intra-abdominal infection
Cmax	Peak plasma concentration
CPE	Carbapenemase-producing Enterobacterales
CRE	Carbapenem-resistant Enterobacterales
CT	Computed tomography
ESBL	Extended-spectrum beta-lactamase
ESICM	European Society of Intensive Care Medicine
ESMID	European Society of Clinical Microbiology and Infectious Diseases
fT	Dosing interval
IAH	Intra-abdominal hypertension
IAI	Intra-abdominal infection
IAP	Intra-abdominal pressure
ICU	Intensive care unit
IDSA	Infectious Diseases Society of America
KPC	Klebsiella pneumoniae carbapenemase
MAP	Mean arterial pressure
MBLs	Metallo-beta-lactamases
MDR	Multi-drug resistance
MIC	Minimum inhibitory concentration
PCT	Procalcitonin
PD	Pharmacodynamic
PDR	Pan-drug resistance
PK	Pharmacokinetic
SOFA	Sequential Organ Failure Assessment
TDM	Therapeutic drug monitoring
US	Ultrasound
VRE	Vancomycin-resistant Enterococcus faecium
XDR	Extensively drug resistance
WISS	WSES complicated IAIs Score Study
